# A pre-trained BERT for Korean medical natural language processing

**DOI:** 10.1038/s41598-022-17806-8

**Published:** 2022-08-16

**Authors:** Yoojoong Kim, Jong-Ho Kim, Jeong Moon Lee, Moon Joung Jang, Yun Jin Yum, Seongtae Kim, Unsub Shin, Young-Min Kim, Hyung Joon Joo, Sanghoun Song

**Affiliations:** 1 School of Computer Science and Information Engineering, The Catholic University of Korea, Bucheon, Republic of Korea; 2 Korea University Research Institute for Medical Bigdata Science, Korea University, Seoul, Republic of Korea; 3 Department of Biostatistics, Korea University College of Medicine, Seoul, Republic of Korea; 4 Department of Linguistics, Korea University, Seoul, Republic of Korea; 5 Department of Cardiology, Cardiovascular Center, Korea University College of Medicine, Seoul, Republic of Korea; 6 Department of Medical Informatics, Korea University College of Medicine, Seoul, Republic of Korea; 7 School of Interdisciplinary Industrial Studies, Hanyang University, Seoul, Republic of Korea

**Keywords:** Biomedical engineering, Computer science

## Abstract

With advances in deep learning and natural language processing (NLP), the analysis of medical texts is becoming increasingly important. Nonetheless, despite the importance of processing medical texts, no research on Korean medical-specific language models has been conducted. The Korean medical text is highly difficult to analyze because of the agglutinative characteristics of the language, as well as the complex terminologies in the medical domain. To solve this problem, we collected a Korean medical corpus and used it to train the language models. In this paper, we present a Korean medical language model based on deep learning NLP. The model was trained using the pre-training framework of BERT for the medical context based on a state-of-the-art Korean language model. The pre-trained model showed increased accuracies of 0.147 and 0.148 for the masked language model with next sentence prediction. In the intrinsic evaluation, the next sentence prediction accuracy improved by 0.258, which is a remarkable enhancement. In addition, the extrinsic evaluation of Korean medical semantic textual similarity data showed a 0.046 increase in the Pearson correlation, and the evaluation for the Korean medical named entity recognition showed a 0.053 increase in the F1-score.

## Introduction

In the field of clinical NLP, the medical history of patients, diagnosis, and treatment information are crucial for text processing. Therefore, word-level language representation models such as BioWordVec^1^ have been developed and studied using Word2Vec^2^ and Fasttext^3^. However, medical text is extremely challenging to analyze because of the complexity of word representations and jargon. Therefore, the adoption of a domain-specific approach is required for the development of deep-learning-based language models in medical text processing.

With significant progress in deep learning over the past few years, natural language processing (NLP) models have been developed to overcome the limitations of existing models used for language processing. Bidirectional encoder representations from transformers (BERT) is a deep learning language model constructed using the encoder structure from the transformer^4^. BERT has shown impressive performance in various NLP tasks and has revolutionized the NLP domain. Numerous language models are being developed using BERT^5–7^.

BERT is a pre-trained language model that focuses on English. Furthermore, a multi-linguistic version of BERT architecture can be developed by modifying the vocabulary of the model and using relevant data used for training^4^. However, this approach does not sufficiently reflect various properties of a language^8^. Therefore, language-specific BERT has been studied in various languages. The Korean-based BERT (KR-BERT) is a representative language-specific model of the Korean language^9^. This model has shown remarkable results and excellent performance compared with other models trained using the Korean corpus. However, the model still has a chance to improve language understanding for text analysis in the specialized domain when the existing domain-specific English models are considered^10–13^. Domain-specific BERT models have been studied in the medical, financial, and legal fields. In particular, BioBERT showed a performance increase compared to the existing BERT model by training the model with the biomedical corpus^10^. This demonstrates the requirement for domain-specific training for BERT.

In this paper, we describe the Korean Medical BERT (KM-BERT) model that has been trained for the Korean medical corpus from the existing BERT model by combining language-specific and domain-specific approaches. To demonstrate the validity of the KM-BERT model, we performed intrinsic and extrinsic evaluations on a Korean medical text dataset. The results demonstrate the effectiveness of KM-BERT in comparison with existing language models.

## Related works

### BERT

BERT performs two unsupervised pre-training tasks to improve the language understanding of machines. The pre-training tasks consisted of a masked language model (MLM) and next sentence prediction (NSP) using an unlabeled corpus (800M words from BookCorpus and 2500M words in English from Wikipedia). BERT used a WordPiece tokenizer composed of 30,522 subwords^14^.

BERT adopts the encoder structure of a deep learning model, transformers, which consists of the encoder and decoder^15^. BERT architecture is composed of bidirectional encoders, and the structure is constructed by stacking the encoders. BERT architecture uses three types of embeddings as input. These are token embeddings, position embeddings, and segment embeddings. After the input layer, encoder layers with multi-head attention based on the scaled dot-product. Finally, a fully-connected layer is added to the last layer of the stacked encoders. When BERT performs fine-tuning for a specific NLP task, such as question-answering, the fully-connected layer is replaced with the proper layer.

Similarly, multilingual BERT (M-BERT) uses an identical network structure and two types of pre-training methods. However, M-BERT uses different data sources, such as Wikipedia, in the top 104 languages, including English. To cover a wide range of languages as a single model, M-BERT uses a shared word-piece tokenizer with the vocabulary size of 110K.

### Korean-specific language model

M-BERT has demonstrated unparalleled optimization for NLP tasks using various languages. However, language-specific BERT models generally outperform multilingual models^8,16^ because the multilingual models are hard to consider linguistic properties of the local language separately. KR-BERT^9^ is a state-of-the-art language-specific NLP model developed for Korean. It adopts a BidirectionalWordPiece tokenizer based on forward and backward byte-pair encoding for subword segmentation. The vocabulary of the KR-BERT tokenizer contains 16,424 subwords, reflecting the different properties of the Korean language. KR-BERT can therefore enhance the Korean language understanding of the model and has shown a slightly improved performance compared to other existing Korean NLP models such as KoBERT and KorBERT (not published).

### Limitations of KR-BERT in the medical domain

Despite the suitable performance of the Korean-specific BERT model, it has several limitations in terms of its application in the medical domain, where incorrectly tokenized words are more frequently observed than in common contexts. For example, the medicine ‘ibuprofen’ should be recognized as a single token, but it is tokenized into ‘i’, ‘##bu’, ‘##pro’, ‘##fen’ (‘ib’, ‘##up’, ‘##ro’, ‘##fen’ in case of English). This type of incorrect tokenization can degrade the language understanding of a model. Furthermore, domain-specific medical terminology makes understanding the context difficult even for humans, owing to its unfamiliarity. These terms are used intermittently, and training language models in an adequate manner is challenging. To resolve these problems, the training of language models using domain-specific corpora is required.

## Methods

### Dataset

Three types of Korean medical documents were collected. Clinical research articles were selected from domestic scholarly journals published in Korean. Health information news articles were selected from well-known newspapers targeting common readers with no expert knowledge. Medical textbooks were selected at an intermediate level because they introduce expert knowledge to medical students, and the documents were well-structured and of very high quality^17^. (1) Medical textbooks: Two Korean publishing companies provided textbooks for this study. Each publisher had a classification system for the subject of books, and textbooks from 54 subfields (e.g., internal medicine, emergency medicine, orthopedics, dentistry, pharmacy, and public health) of the medical field were selected. Finally, 518 text files from Korean medical textbooks, published between 2010 and 2020, were used. (2) Health information news: NAVER, a widely used internet portal site in Korea, distributes news articles from general newspapers, internet newspapers, and broadcasting stations. We collected all news articles from a section called ‘Health Information’ in the ‘News’ section of the NAVER portal. Approximately 72,844 health information news articles published from January 1, 2010, to December 31, 2020, in NAVER were collected through Internet crawling. (3) Medical research articles: 72 journals (e.g., Journal of the Korean Society of Integrative Medicine, Journal of the Korean Society of Emergency Medicine) published in Korean were selected from the journals listed in the Korean Studies Information Service System, and 15,698 medical research articles published between 2010 and 2020 were collected. The text files were parsed and modified for further investigation. Consequently, we were able to build a Korean medical corpus consisting of approximately six million sentences and 116 million words.

### Korean medical language model

We present KM-BERT trained on the medical corpus to overcome the aforementioned limitations. We adopted the KR-BERT character tokenizer and the pre-training framework used in BERT to train KM-BERT for medical language understanding. For efficient training, the initial weights of KM-BERT were replaced with the weights of the pre-trained KR-BERT, rather than starting from scratch.

**Figure 1:**
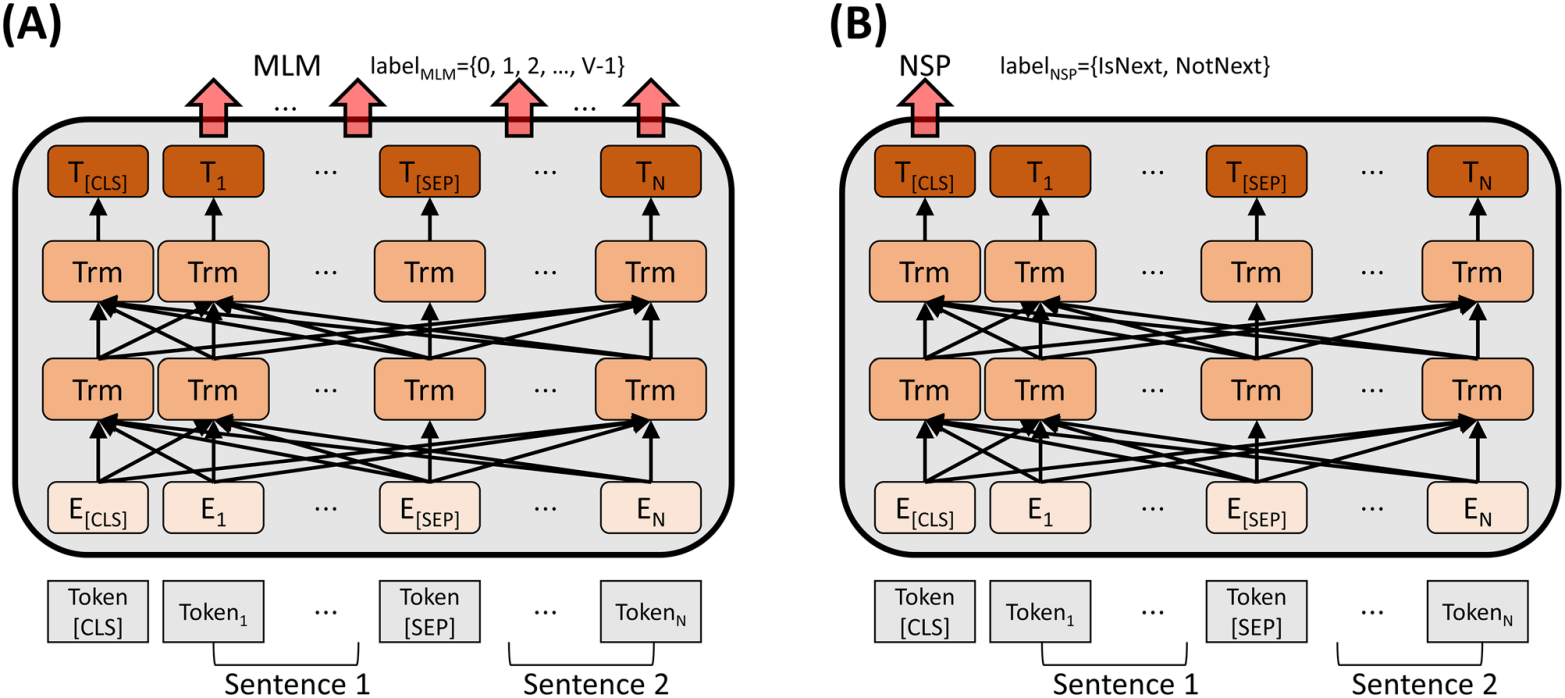
Pre-training process for KM-BERT. A pair of sentences joined by two sentences with special tokens is used as input. (**A**) MLM task. (**B**) NSP task.

In the pre-training process, KM-BERT was trained for the MLM and NSP tasks following the BERT strategy (Fig. 1). The MLM is a token-level task only for masked tokens, and the NSP determines whether two sentences have a prior relationship. Both tasks aim to classify the correct label by minimizing the cross-entropy loss of the model for two sentences.

In the MLM, 15% of all tokens were randomly masked for each sentence, and only the masked tokens were considered for prediction. Among the masked tokens, 80% were replaced with the [MASK] token, 10% with a random token, and the remaining 10% were unchanged. The labels that masked the tokens were limited by the size of the vocabulary. In other words, the size of the MLM labels corresponded to the entire vocabulary size of the model. Let *c* be a one-hot label vector for a masked token and *p* be the predicted probability. The MLM loss for a single masked token can be defined as

$$ loss_{MLM} = - \mathop \sum \limits_{i = 0}^{V - 1} c_{i} \log \left( {p_{i} } \right), $$

where *V* denotes the vocabulary size. We used the average MLM loss for all masked tokens in a paired sentence.

The NSP contains two labels: *IsNext* and *NotNext*. Let *c*_*IsNext*_ and *p*_*IsNext*_ be the label and predicted probability of the next sentence relationship, respectively. The NSP loss can be defined as follows:

$$ loss_{NSP} = - c_{IsNext} \log \left( {p_{IsNext} } \right) - \left( {1 - c_{IsNext} } \right)\log \left( {1 - p_{IsNext} } \right). $$

Subsequently, the total loss can be derived by the summation of both losses, and the weights of the model are updated over the total loss.

### Extended vocabulary

Frequently, incorrect tokenization may interfere with language understanding. To reduce the number of these cases, we supplemented the vocabulary using a tokenizer. We collected 70,415 medical terms by crawling the medical terminology databases released by the Korean Association of Medical Journal Editors (KAMJE) (53,511 entries) and the Ministry of Food and Drug Safety (MFDS) (16,904 entries). We considered 69,206 medical terms by eliminating terms that overlapped between the two databases. Although the terms in the KAMJE and MFDS are potentially informative or meaningful depending on the context, several words are rarely used, even in medical corpora. To eliminate rare words, we considered the number of appearances of each term in the Korean medical corpus. We considered the terms of the main ingredients and additives from the MFDS drug datasets and removed words that appeared less than three times in the entire corpus. From these, we selected the top 10,000 words that were frequently used in the Korean medical corpus. In addition, 1629 words were added from the similarity and relatedness experiments and merged into the extended vocabulary. The details of the similarity and relatedness experiments are described in the previous work^17^.

Finally, 10,562 medical terms were obtained, excluding duplicates with the KR-BERT vocabulary. To improve linguistic understanding in the medical domain, we adopted this extended medical vocabulary of 26,986 words for KM-BERT (KM-BERT-vocab). The weight of the token embeddings for KM-BERT-vocab was initialized by normalization.

It was expected that through training the KM-BERT-vocab model, the model’s understanding of the medical field language would improve. For instance, the Korean Romanization of ‘systemic sclerosis’ is ‘censinkyenghwacung’ and that of ‘nephrosclerosis’ is ‘sinkyenghwacung’. Both these Korean terms are comprised of the same root morpheme or lexical head, and the meaning difference is because of the prefix 'cen', which is dependent on the head. Therefore, the morphological complexity of medical terms is critical to the performance of tokenizers. The extended vocabulary contains both the medical terms that are tokenized into their corresponding single tokens. KR-BERT could not appropriately tokenize the corresponding terms into ‘cen’, ‘sin’, ‘kyeng’, ‘hwa’, ‘cung’ tokens and ‘sinkyeng’, ‘hwa’, ‘cung’ tokens. It must be noted that ‘sinkyeng’ means nerve in Korean. This example suggests that including a supplement to medical vocabulary can be beneficial for NLP in professional and complex medical fields.

Both KM-BERT and KM-BERT-vocab are available at https://github.com/KU-RIAS/KM-BERT-Korean-Medical-BERT.

## Results

Through NLP experiments, we evaluated the language understanding capability of KM-BERT and compared its performance with that of other language models. The M-BERT and KR-BERT models were considered as baseline models in the experiments.

### Experiments

We performed pre-training, two types of intrinsic evaluation, and two types of extrinsic evaluation. The Korean medical corpus was used for pre-training; the corpus was randomly split into a training set of 90% and a validation set of 10%. The model was trained on the training set and its performance was measured using a validation set.

Additionally, we collected an external dataset to intrinsically evaluate the models. The dataset comprised three sets of 294 sentence pairs, with the next sentence relationship. Sentence pair examples in each set were extracted from medical textbooks, health information news, and medical research articles after manual inspection for errors, such as encoding failures. We selected informative sentences by manual investigation to exclude meaningless sentences, such as sentences that were extremely short and only human names. However, there was no overlap between the Korean medical corpus and the external dataset. The medical textbooks used in the external dataset were not from the two mentioned publishers. In addition, we only considered health information news uploaded in January and February 2021 and medical research articles published in 2009.

Finally, we acquired the Korean medical semantic textual similarity (MedSTS) dataset, wherein each sentence was translated from the original MedSTS, which consisted of 3121 English sentence pairs and a corresponding similarity score of 0–5^18^. First, we translated each English sentence in the MedSTS dataset into Korean using the Python library of Google Machine Translation. We manually reviewed each translation result and refined the mistranslation and low-quality translation results. During this process, the similarity scores for each sentence pair did not change. We used 2393 sentence pairs for the training set and 728 for the test set^19,20^. We also acquired the Korean medical named entity recognition (NER) dataset consisting of 2189 Korean medical sentences with tagged clinical terminology^21^. We used 5-fold cross validation to evaluate the medical tagging performance of each model. Table 1 shows an overview of the evaluation and datasets used.

**Table 1:** Description for datasets used in evaluations.

Task	Evaluation	Data description
MLM and NSP	Pre-training	Test set of the collected corpus
MLM	Intrinsic	External Korean medical text
NSP	Intrinsic	External Korean medical text
MedSTS	Extrinsic	MedSTS dataset translated from English to Korean
NER	Extrinsic	Korean medical NER dataset

All the experiments were performed on an Ubuntu 18.04.2 LTS server with two Intel (R) Xeon (R) Silver 4210R CPU 2.40 GHz, 256 GB RAM, and dual GPUs of RTX 3090.

### Pre-training

The collected Korean medical corpus was used for the pre-training of BERT for the MLM and NSP tasks. The MLM task aims to predict an appropriate token label for each masked token, and the NSP task performs classification using two labels (*IsNext* or *NotNext*). Each task follows a supervised mechanism during the learning process. However, the required data can be constructed from an unlabeled corpus by randomly joining two sentences and masking the token. Half of the sentence pairs were replaced with two irrelevant sentences. In other words, the ratio of *IsNext* to *NotNext* labels was one-to-one for the NSP task. Next, the sentences were tokenized into tokens, and each token was randomly masked for the MLM task. Therefore, we built a six-million sentence pair dataset for pre-training based on the Korean medical corpus.

Considering the computational specifications, we used a batch size of 32 and a maximum sentence length of 128 tokens. In addition, we used a learning rate of 1e−6.

**Figure 2:**
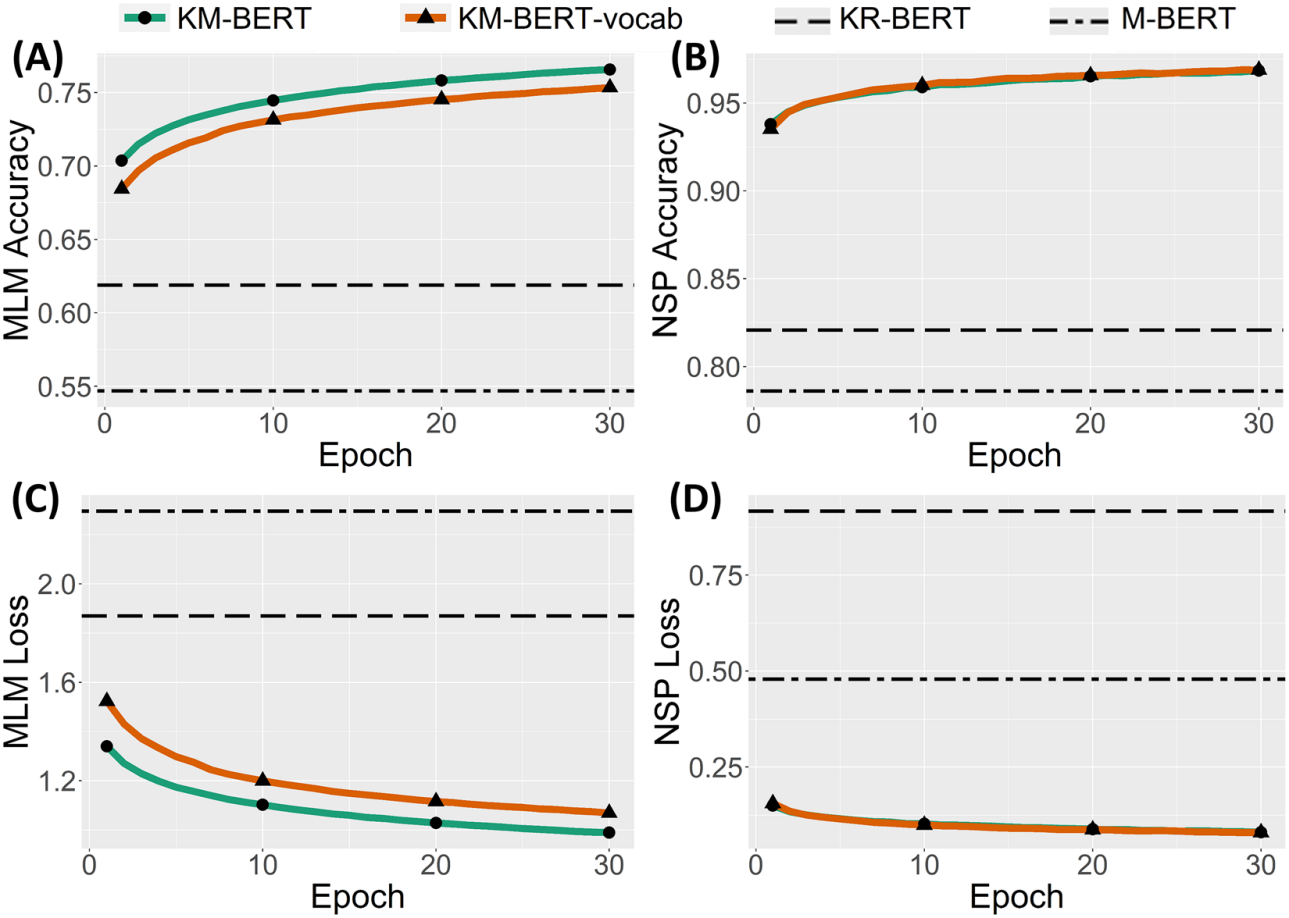
Pre-training results of KM-BERT and KM-BERT-vocab for MLM and NSP tasks over epoch. Dashed line representing KR-BERT and dot-dashed line representing M-BERT denotes the performance of the final pre-trained models. (**A**) MLM accuracy. (**B**) NSP accuracy. (**C**) MLM loss. (**D**) NSP loss.

The change in performance during the pre-training process over the epochs is depicted (Fig. 2). For comparison with the baseline language models, we measured the performance of pre-trained KR-BERT and M-BERT over the validation set. M-BERT achieved an MLM accuracy of 0.547, an NSP accuracy of 0.786, an MLM loss of 2.295, and an NSP loss of 0.479. KR-BERT achieved an MLM accuracy of 0.619, an NSP accuracy of 0.821, an MLM loss of 1.869, and an NSP loss of 0.916. KR-BERT showed slightly better performance than M-BERT, except for the NSP loss. Both KM-BERT and KM-BERT-vocab showed improved performance compared to the baseline language models. A performance gap appears even after one training epoch. This implies that training with domain-specific medical corpora enhances language understanding of medical texts.

### Intrinsic evaluation

Intrinsic evaluation was performed for MLM and NSP on external Korean medical text that consists of medical textbooks, health information news, and medical research articles to compare the language understanding capability of the model.

The MLM task was performed on three sets of 294 sentence pairs. In this evaluation, identical rules were used to mask the tokens. The rules contained random aspects. Thus, performance was measured using 100 repetitions of the MLM task.

**Figure 3:**
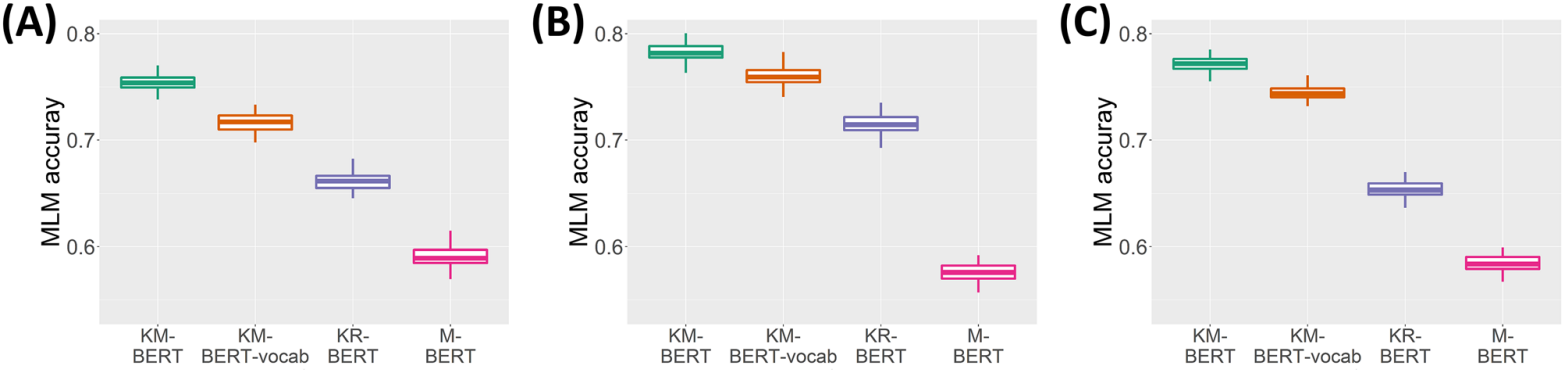
Distribution of MLM accuracy by 100 repetitions for each language model over each corpus type. (**A**) Medical textbook. (**B**) Health information news. (**C**) Medical research article.

The MLM accuracy of each language model for the external Korean medical corpus was evaluated through repeated experiments (Fig. 3). KM-BERT outperformed the pre-trained language models on MLM and KM-BERT-vocab, regardless of the corpus type. M-BERT exhibited the lowest performance. The performance of the four identical models can vary depending on the type of corpus used. Except for M-BERT, the overall performance of the models was higher for health information news than for other medical textbooks and medical research articles. Considering that medical textbooks and research articles are specialized and difficult to decipher, it can be inferred that these models performed better for general and popular health information news. This suggests that the development of domain-specific NLP models is necessary, highlighting the effectiveness and urgent requirements of pre-trained models (KM-BERT and KM-BERT-vocab). Furthermore, the difference in the performance on health information news between KM-BERT and KR-BERT was 0.067, whereas it was 0.119 for medical research articles.

Additionally, we performed the NSP task on the same external dataset used in the MLM task. For the NSP, we generated three additional sets of 294 random sentence pairs with no next relationship. In other words, for each type, there were 294 sentence pairs that needed to be classified as next sentence relationships and 294 random sentence pairs that should not be.

**Figure 4:**
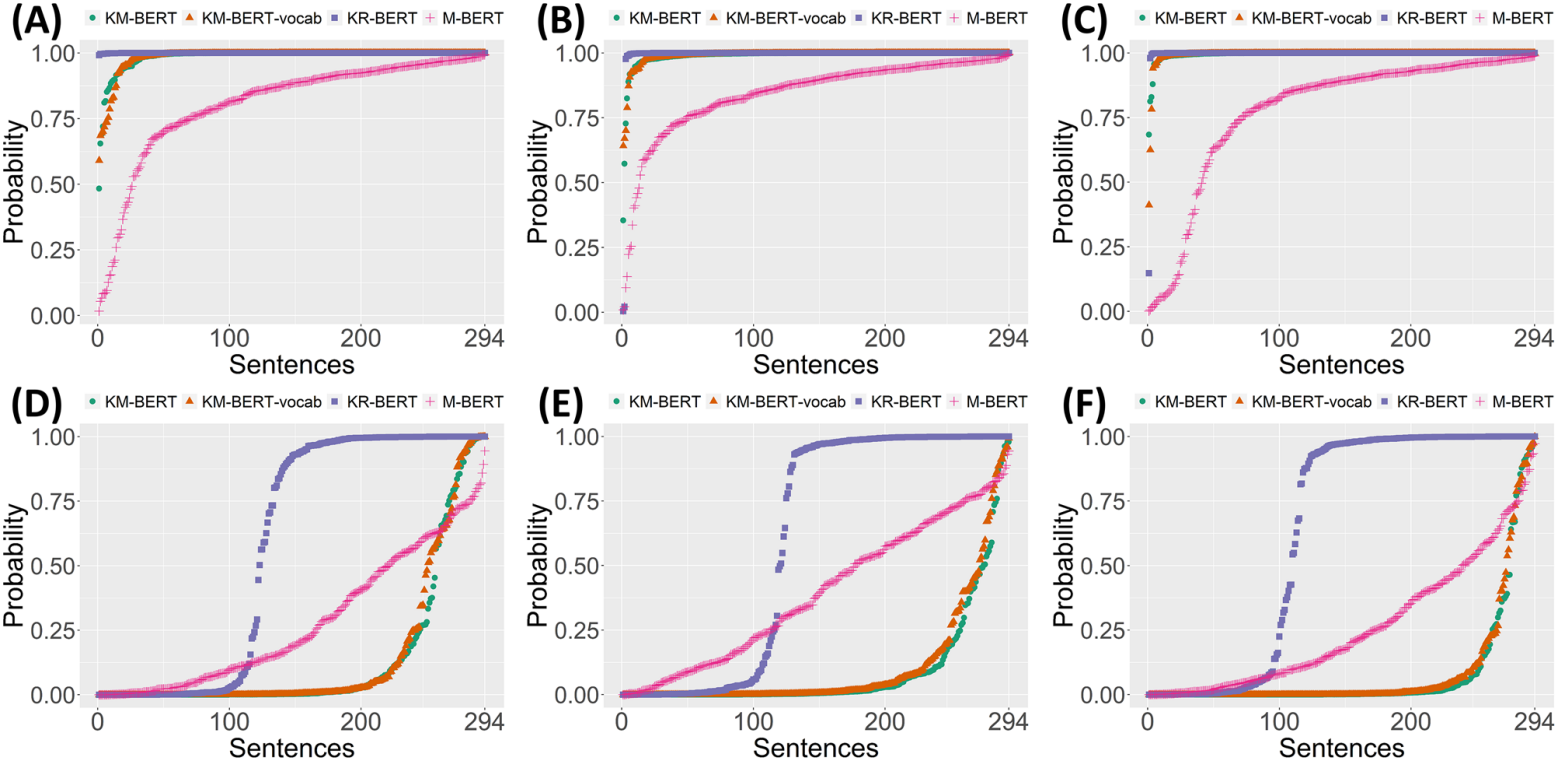
Distribution of the predicted next sentence probability for the NSP task. (**A**–**C**) Medical textbook, health information news, and medical research article with the next sentence relationship. (**D**–**F**) Random sentence pairs for corpus types that correspond to (**A**–**C**) with no next sentence relationship.

We measured the predicted probability for the NSP sorted in increasing order for each model (Fig. 4). Each model classified three groups of next sentence pairs for medical textbooks, health information news, and medical research articles. All samples in the three groups were constructed to have the next sentence relationship. The remaining three groups consisted of random sentence pairs for medical textbooks, health information news, and medical research articles. Overall, NSP performance was high in the next sentence groups (Fig. 4A–C). These four models showed error rates of less than 10% for binary classification over the next relationship. By contrast, NSP performance was lower in data groups with no next sentence relationships (Fig. 4D–F). KR-BERT showed a considerably large error for the *NotNext* label compared with an extremely low error for the *IsNext* label. Despite this degradation, KM-BERT and KM-BERT-vocab showed relatively low errors for the *NotNext* label compared to the other models. The results clearly show that domain-specific pre-training can influence language understanding of the corresponding domain corpus.

This overall tendency coincides with the results of previous studies on the effects of pre-training using an unsupervised NSP task. It has been reported that BERT representations become increasingly sensitive to discourse expectations, such as conjunction and negation, in biomedical texts when BERT architecture is further pre-trained on biomedical corpora using the NSP training objective^22^. Specifically, BioBERT trained on PubMed articles and abstracts showed some improvements in understanding the underlying discourse relations suggested by the Biomedical Discourse Relation Bank^23^.

The details of NSP accuracy shown in Fig. 4 are presented (Table 2). The NSP accuracy was evaluated by determining the next sentence relationships with a predicted probability of 0.5, as the threshold. KM-BERT-vocab showed the highest NSP accuracy among the groups with the next sentence relationships. In the same group, M-BERT exhibited the lowest NSP accuracy. The gap in NSP accuracy by model was greater in data groups that had no next relationship than in data groups with the next relationship. The language model with the best NSP accuracy was KM-BERT. KM-BERT achieved slightly higher NSP accuracy than KM-BERT-vocab. KR-BERT showed the lowest accuracy in the same data group, with performance differences compared with KM-BERT. This can be interpreted as a limitation in the sentence relation inference of KR-BERT for medical domain texts.

**Table 2:** NSP accuracy of the intrinsic evaluation dataset.

Model	Medical textbooks		Health information news		Medical research article		Overall
	*IsNext*	*NotNext*	*IsNext*	*NotNext*	*IsNext*	*NotNext*	
KM-BERT	0.997	0.871	0.997	0.932	1	0.935	0.955
KM-BERT-vocab	1	0.850	1	0.922	0.997	0.925	0.949
KR-BERT	1	0.415	0.993	0.408	0.997	0.371	0.697
M-BERT	0.912	0.752	0.956	0.599	0.861	0.810	0.815

### Extrinsic evaluation

Extrinsic evaluations were performed for the MedSTS dataset and the Korean medical NER dataset to demonstrate the performance of fine-tuning for downstream tasks. We investigated the Pearson correlation and Spearman correlation between the similarity measured by each language model and the similarity measured by human verification of MedSTS. We measured the F1-score for the tagging task of the Korean Medical NER dataset. Each model was fine-tuned using the training set, and their performance was evaluated using the test set. We used a batch size of 32 and considered learning rates of 2e−5, 3e−5, and 5e−5, and training epochs of 2, 3, and 4.

### Korean MedSTS task

For the MedSTS task, the best performance of each language model trained using hyperparameter candidates is presented (Table 3). The best-performing language model for the sentence similarity measurement task was KM-BERT. By contrast, KR-BERT showed the lowest measured correlation with the predicted sentence similarity. This indicates that the sentence relationship in the MedSTS dataset was properly trained through pre-training on Korean medical corpora.

**Table 3:** Extrinsic evaluation results on MedSTS.

Model	Pearson correlation	Spearman correlation
KM-BERT	0.869	0.860
KM-BERT-vocab	0.851	0.834
KR-BERT	0.823	0.811
M-BERT	0.842	0.830

We explored two cases of similarity measurements using KM-BERT and KR-BERT with examples from the MedSTS dataset. Two cases of sentence pairs that showed performance differences in sentence similarity measured in each model are presented (Table 4). In the above example, the similarity score predicted by KM-BERT was comparable to the similarity measured by human experts. This is probably because the embeddings for the drugs and formulations are different between KM-BERT and KR-BERT. The bottom is a case in which KR-BERT measures the similarity closer to the human score. This example is related to the general instruction of patient care in the management of medical systems or hospitals, and therefore, it may not require expert knowledge to understand the instructions.

**Table 4:** Examples of the MedSTS dataset containing the true MedSTS similarity and similarities measured by KM-BERT and KR-BERT.

Sentences		Similarity		
Sentence 1	Sentence 2	Med STS	KM-BERT	KR-BERT
Qsymia 3.75–23 mg capsule multiphasic release 24 h 1 capsule by mouth one time daily	Aleve 220 mg tablet 2 tablets by mouth one time daily as needed	0	1	3
Patient requires extensive assistance in the following activities: toileting, transfer to/from bed/chair, mobility	Patient requires limited assistance in the following activities: bathing, dressing, toileting	2.75	4	3

Sentences were translated from the original English MedSTS sentences into Korean.

### Korean Medical NER task

In addition to the MedSTS task, we evaluated the pre-trained model using the Korean Medical NER dataset. The dataset was composed of three medical tags: body parts, diseases, and symptoms. The performance of the Korean medical NER was measured using the averaged F1-score for the three medical tags (Table 5). KM-BERT showed the highest F1-score with a performance gap of 0.019. A performance increase was observed in comparison with KR-BERT and M-BERT. This implies that pre-training in the Korean medical corpus is effective for Korean medical NER tasks.

**Table 5:** Extrinsic evaluation results on Korean medical NER.

Model	F1
KM-BERT	0.866
KM-BERT-vocab	0.847
KR-BERT	0.847
M-BERT	0.813

## Discussion

In this study, we presented a pre-trained Korean medical NLP model. The pre-trained model, KM-BERT, was trained on the Korean medical corpus collected from three types of articles (medical textbooks, health information news, and medical research articles). The Korean medical corpus consists of six million unlabeled sentences and 116 million words. KM-BERT was used to pre-train the language understanding of the medical domain written in Korean using an unlabeled corpus for the MLM and NSP tasks.

To demonstrate the superiority of KM-BERT, particularly in the Korean medical domain, we performed intrinsic and extrinsic evaluations. We investigated the performance of the pre-trained model and baselines for a new Korean medical corpus and the MedSTS dataset. The results demonstrated the superior performance of the pre-trained language model compared with the baseline language models. The KM-BERT model showed an overall improved capability for language understanding.

### Language-specific perspective

M-BERT is an advanced version of BERT; however, its performance is inferior to that of language-specific models. The Dutch BERT model (BERTje) and the Estonian BERT model (EstBERT) were developed to overcome the inferior language representations of the multilingual model^8,24^. Martin et al. suggested a monolingual language model using a small-corpus dataset^16^. Lee et al. proposed a small-scale Korean language model by employing a BidirectionalWordPiece tokenizing method^9^. Although these models were able to reflect some aspects of the language structure of Koreans, domain-specific medical knowledge was not simultaneously considered.

Despite the great transferability of M-BERT across languages, it has been proven that language-specific BERT models perform better than M-BERT. Pires et al. argued that M-BERT cannot learn systematic transformations of language structures and, therefore, cannot accommodate a target language with different word orders^25^. The order of words in Korean is also different from that in Indo-European languages such as English and French. Specifically, because of the well-developed case-marking system in Korean, sentences with different word orders can deliver the same meaning by marking the semantic role, for example, the agent or patient of nouns. Therefore, a specialized BERT model must be used for the Korean language.

### Domain-specific perspective

It has been suggested that domain-specific BERT embeddings such as SciBERT^13^ and LEGAL-BERT^11^ outperform BERT in several tasks that require an understanding of specialized terms and usage. For instance, LEGAL-BERT showed improvements in predicting the labels of legal terms that specify the details of commercial contracts and legal cases. Similarly, SciBERT becomes more accurate in recognizing clinical entities and scientific concepts when BERT architecture is pre-trained using journal articles and abstracts. These empirical findings confirm the efficacy of the domain-adaptation of BERT for capturing the linguistic structures of legal texts, scientific articles, and clinical narratives that are scarcely observed in generic corpora.

Lee et al. proposed BioBERT, which is a representative pre-trained model specialized in textual analysis in the biomedical domain^10^. Because BioBERT uses an identical tokenizer, there is insufficient consideration for professional medical terminology. Huang et al. developed a ClinicalBERT model of clinical notes in hospitals^12^. ClinicalBERT focuses on readmission prediction rather than on general usage.

### Limitations

The present study has several limitations. First, there are limitations in terms of the computational power and data resources. In contrast to the current deep learning trends based on large-scale computing, the pre-training task in this study was performed using a common computational resource, incurs excessive cost, and is time-consuming (approximately one month). Nevertheless, this type of deep learning research is believed to be advantageous because it can be conducted in a single laboratory without the use of large-scale computing resources. Second, KM-BERT was not pre-trained from scratch. KM-BERT used the initial weights from KR-BERT. The pre-training approach adopted in this study is expected to help develop language-specific models for domain-specific models. However, it has to be stated that if the model had been pre-trained from scratch and then used for comparison and analysis, more meaningful results may have been obtained. Third, the advantage of applying an extended medical vocabulary, KM-BERT-vocab, to a language model is unclear. The KM-BERT-vocab showed a slight increase in accuracy in the NSP evaluation of *IsNext* label; however, it did not improve performance in the other downstream tasks.

Although there was no performance deterioration owing to the embedding layer weight initialization for KM-BERT-vocab compared with existing models, KM-BERT showed better performance than KM-BERT-vocab. Extended vocabulary can help the model handle medical terminology as it is but can decrease the model performance because we initialized the weight of the embedding layer. Therefore, careful strategies for selecting extended vocabulary are required to improve a language model by supplementing its vocabulary. Furthermore, another downstream task study is needed to elucidate the benefits of KM-BERT-vocab. In future studies, the effectiveness of KM-BERT will be validated via word vector representation in a specialized medical context, and the collection of Korean medical text will be thoroughly designed based on appropriate data statements^26^.

## Conclusions

The present study demonstrates the feasibility of domain-specific pre-training using a pre-trained language-specific model. This study demonstrated that the language understanding of the model improved when the pre-learning approach was used. Furthermore, the intrinsic evaluation demonstrated the language understanding capability of the model, and the extrinsic evaluation demonstrated its applicability to other NLP tasks. KM-BERT showed the best performance for the pre-training, intrinsic, and extrinsic evaluations in this study. The results show the feasibility of pre-training for domain-specific text using a monolingual language model rather than an English model. We expect that KM-BERT will be beneficial to Korean practitioners in the medical domain. In the future, we expect our approach to be applied to the development of language models specialized for specific domains in a variety of other languages.

## Data Availability

The datasets generated and/or analysed during the current study are not publicly available due to not public text sources but are available from the corresponding author on reasonable request.
