# Author Correction: A pre-trained BERT for Korean medical natural language processing

**DOI:** 10.1038/s41598-023-36519-0

**Published:** 2023-06-07

**Authors:** Yoojoong Kim, Jong-Ho Kim, Jeong Moon Lee, Moon Joung Jang, Yun Jin Yum, Seongtae Kim, Unsub Shin, Young-Min Kim, Hyung Joon Joo, Sanghoun Song

**Affiliations:** 1 School of Computer Science and Information Engineering, The Catholic University of Korea, Bucheon, Republic of Korea; 2 Korea University Research Institute for Medical Bigdata Science, Korea University, Seoul, Republic of Korea; 3 Department of Biostatistics, Korea University College of Medicine, Seoul, Republic of Korea; 4 Department of Linguistics, Korea University, Seoul, Republic of Korea; 5 Department of Cardiology, Cardiovascular Center, Korea University College of Medicine, Seoul, Republic of Korea; 6 Department of Medical Informatics, Korea University College of Medicine, Seoul, Republic of Korea; 7 School of Interdisciplinary Industrial Studies, Hanyang University, Seoul, Republic of Korea

Correction to: *Scientific Reports* https://doi.org/10.1038/s41598-022-17806-8, published online 16 August 2022

The original version of this Article contained errors in the Author Information section.

“These authors contributed equally: Yoojoong Kim, Jong-Ho Kim, Hyung Joon Joo and Sanghoun Song.”

now reads:

“These authors contributed equally: Yoojoong Kim and Jong-Ho Kim.

These authors jointly supervised this work: Hyung Joon Joo and Sanghoun Song.”

Additionally, Hyung Joon Joo was omitted as a corresponding author. Correspondence and requests for materials should also be addressed to drjoohj@korea.ac.kr.

The original Article has been corrected.

